# Factors influencing the negative emotions of undergraduate medical students

**DOI:** 10.3389/fpsyg.2025.1653246

**Published:** 2025-09-15

**Authors:** Chao Li, Weiju Gu

**Affiliations:** ^1^Henan Medical University, Xinxiang, Henan, China; ^2^The Third Clinical College of Henan Medical University, Xinxiang, Henan, China

**Keywords:** influencing factors, medical students, mental health, negative emotions, strategies

## Abstract

The distinct nature of medical education predisposes undergraduate medical students to psychological challenges and in particular negative emotions such as anxiety, depression, and loneliness. The development of negative emotions in these students results from the interplay of multiple factors, mainly including academic overload, interpersonal dynamics, and psychological resilience. Yet, researches on this topic have predominantly used cross-sectional designs and have often relied on samples from a single cultural context, thereby restricting the generalizability of the findings. Therefore, future researches should (i) prioritize longitudinal studies to track the dynamic evolution of negative emotions, (ii) investigate coping strategies for emotion regulation, and (iii) conduct cross-cultural comparisons to identify key influencing factors. In terms of coping strategies, layered interventions can be implemented to incorporate cognitive behavioral strategies to enhance emotion regulation, and a recommendation is to reform training programs to alleviate the overall pressure on medical undergraduates.

## 1 Introduction

The demanding nature and intrinsic professional demands of medical training render undergraduate medical students particularly susceptible to psychological comorbidities. As a distinct demographic, these students consistently face a range of challenges including academic overload, professional responsibilities, and complex interpersonal dynamics (Agyapong-Opoku et al., 2023; Fauzi et al., 2021; Alshareef et al., 2024). The heightened incidence of negative emotions in this group has become a significant issue in medical education worldwide, and a thorough understanding of the prevalence and determinants of negative emotions among medical undergraduates is essential for developing effective interventions and support mechanisms designed to improve their academic performance and overall wellbeing.

Negative emotions encompass distressing affective states such as anger, fear, sorrow, and anxiety, which frequently result in psychological discomfort (Pekrun et al., 2007). The influence of negative emotions on medical undergraduates is substantial; these emotions not only impair academic performance but may also adversely affect their professional trajectories. Research indicates that enduring negative emotions can result in suboptimal clinical practice performance and potentially influence future career decisions and development (Ishak et al., 2013; Fares et al., 2016; Qu et al., 2022). Consequently, understanding the current state and determinants of negative emotions among medical undergraduates has significant theoretical and practical importance for formulating effective intervention measures and support strategies.

Previous studies on negative emotions were identified through a systematic search of the Web of Science (WOS) database in May 2025. The search strategy employed the following query: TS = (“medical students”) AND TS = (“negative emotions”) AND TS = (“influencing factors”) AND PY = (2010–2025). Inclusion criteria encompassed: empirical studies (cross-sectional, longitudinal, or intervention studies) focusing on medical students/undergraduates, measurement of negative emotions and peer-reviewed publication. Exclusion criteria included studies without original data, samples of non-medical undergraduate students, and literature with unclear research designs. A narrative review was performed to extract themes and analyze content from the included literature, focusing on extracting the categories, mechanisms, and intervention strategies of influencing factors.

## 2 Manifestations and characteristics of negative emotions of medical undergraduates

### 2.1 Main types of negative emotions among medical undergraduates

Negative emotions can be categorized systematically according to their distinct characteristics and manifestations, with primary types including anxiety, depression, loneliness, anger, fear, and shame. In particular, anxiety is characterized by an elevated state of unease or apprehension that can originate from various sources. Among medical undergraduates, test anxiety is a specific form of anxiety that represents a context-specific psychological response elicited by the demands of assessments; this type of anxiety is manifested through physiological stress responses, negative self-evaluation, and an intense fear of academic failure, all of which collectively undermine learning outcomes (Williamson et al., 2024). Empirical research has indicated a high prevalence of test anxiety among medical undergraduates, with reported rates ranging from 52 to 81.1%. Notably, final-year medical undergraduates exhibit the highest levels of test anxiety (Nazir et al., 2021; Loubir et al., 2014). Depression is characterized by symptoms such as low mood and decreased interest, and studies have shown that the incidence of depressive symptoms is elevated among medical undergraduates, particularly during periods of heightened academic pressure (Colonnello et al., 2022). A substantial proportion of medical undergraduates experience pronounced loneliness, attributed to fragmented study schedules, clinical rotations, and the social isolation exacerbated by the COVID-19 pandemic (Keiner et al., 2024; Nashwan et al., 2024). This psychological phenomenon may also be linked to the competitive culture prevalent in medical education, characterized by frequent academic rankings and clinical skill evaluations, which can easily incite self-doubt and social comparison. Academic setbacks or evaluative feedback may provoke emotions such as anger and fear, with anger potentially leading to reduced engagement in educational settings, while fear often stems from anticipatory anxiety about academic failure. Shame is closely tied to self-evaluative processes, wherein medical undergraduates may encounter this emotion during assessments by peers or instructors, thereby undermining their self-efficacy beliefs (Chen X. et al., 2023; Burr and Beck Dallaghan, 2019). A systematic classification of these negative affective states enables the development of targeted psychological interventions aimed at enhancing students' adaptive responses to academic stressors.

### 2.2 Prevalence and severity of negative emotions among medical undergraduates

Survey findings indicate a troubling prevalence of negative emotions among medical undergraduates, with substantial levels of anxiety, depression, and stress reported, particularly during and after the COVID-19 pandemic (Paz et al., 2022; Romic et al., 2021; Lasheras et al., 2020; Sartorao and Sartorao-Filho, 2024). A cross-sectional epidemiological study of Chinese medical undergraduates found that 76.8% of participants exhibited comorbid anxiety–depression symptoms ranging from subthreshold to clinical-grade severity (Chen S. et al., 2023). These findings are particularly concerning given the rigorous nature of medical education, which often intensifies emotional distress. The combination of academic pressure and the emotional demands of medical training contributes significantly to the emotional burden experienced by medical undergraduates, many of whom report moderate to severe anxiety and depression.

### 2.3 Gender and year differences in negative emotion expression

An analysis of gender differences revealed significant disparities in the expression and experience of negative emotions among medical undergraduates. Empirical evidence suggests that female medical trainees exhibit significantly higher levels of affective symptomatology compared to their male counterparts, particularly concerning anxiety–depression comorbidity (Chiou et al., 2021; Mirza et al., 2021). This disparity appears to be mediated by intersecting sociocultural determinants, such as gender-normative expectations and heightened emotional expressivity within female socialization paradigms. Furthermore, the data suggest that the prevalence of negative emotions varies across different academic years: first-year students reported higher levels of anxiety compared to their senior counterparts, likely due to the transition into a challenging academic environment and the associated uncertainties (Chen X. et al., 2023; Loubir et al., 2014). Conversely, another study indicated that senior medical undergraduates exhibited more-pronounced negative emotions, particularly increased fatigue and emotional exhaustion, potentially as a result of prolonged exposure to rigorous medical training (Niemi and Vainiomäki, 2006; Di Vincenzo et al., 2024). The intersection of gender and year of study revealed that female students in their early years were particularly susceptible to anxiety, whereas male students appeared to experience more depressive symptoms as they advanced through their studies (Mocny-Pachońska et al., 2020).

## 3 Factors influencing the negative emotions of medical undergraduates

### 3.1 Relationship between academic burden and negative emotions

Academic pressure is a significant determinant of negative emotional states among medical undergraduates. The academic environment in medical colleges and universities is frequently characterized by elevated levels of stress and pressure, and it can profoundly affect students' emotional wellbeing, resulting in heightened levels of anxiety, depression, and stress. In medical education, students are required to not only acquire extensive professional knowledge but also participate in clinical practice, which undeniably amplifies their academic burden (Tsai and Han, 2023). Research suggests that students subjected to high academic demands often experience feelings of inadequacy and fear of failure, which can intensify negative emotional states. Furthermore, academic stress is positively correlated with test anxiety, indicating that as academic demands increase, so too do the levels of anxiety experienced by students (Sinval et al., 2025). Furthermore, the emotional impact of academic pressure extends beyond immediate stressors, potentially resulting in long-term emotional dysregulation that adversely affects students' overall mental health and academic performance. Also, students' emotional states can influence their learning efficiency, with negative emotions leading to distraction and diminished motivation, thereby creating a vicious cycle (Bista and Chand, 2023). Additionally, the effect of academic burden on negative emotions can be exacerbated by external factors such as social support, personal coping mechanisms, and individual differences in personality traits. Based on Lazarus and Folkman's transactional model of stress and coping, we believe that understanding the complex relationship between academic burden and negative emotions is crucial for developing effective intervention strategies.

Examination anxiety is a widespread concern among students, particularly in high-stakes academic settings. This form of anxiety manifests in various ways, including emotional instability, diminished concentration, and physiological responses such as tachycardia and perspiration. Empirical studies have shown a strong correlation between examination anxiety and students' negative emotional states, with higher anxiety levels amplifying the expression of such emotions. Students experiencing elevated levels of examination anxiety are more prone to report symptoms of depression (Moreira de Sousa et al., 2018; Al Shawi et al., 2018; Memon et al., 2023; Alkharj et al., 2024). Furthermore, examination anxiety has a significant influence on students' learning strategies, often leading to avoidance or procrastination behaviors in the face of impending assessments, thereby intensifying academic stress and negative emotional experiences.

### 3.2 Impact of interpersonal relationships on negative emotions

Interpersonal relationships are pivotal in shaping emotional experiences, particularly concerning negative emotions. We focused on the complex influence of peer support, teacher–student relationships, and social networks. The peer support can substantially mitigate negative emotions and enhance mental health. Medical undergraduates could alleviate anxiety and depression through emotional support from peers during stressful periods (Lasheras et al., 2020; Nomura et al., 2023). Additionally, Lai et al. (2022) found that perceived peer support is inversely correlated with depressive symptoms among university students, suggesting that supportive peer interactions can mitigate feelings of loneliness and anxiety during challenging periods such as the COVID-19 pandemic. The dynamics of peer support extend to the mechanisms of emotion regulation, with individuals who have higher levels of emotional intelligence being more adept at using peer support for this purpose, thereby enhancing their capacity to manage negative emotions (Lopez et al., 2024). This interaction indicates that cultivating environments that promote peer support can substantially improve emotional outcomes for students, particularly those facing academic stress or personal challenges. Furthermore, the impact of peer support is not uniform and may vary with individual differences such as attachment styles and personality traits: students with secure attachment styles are more likely to benefit from peer support, using it effectively to regulate emotions and alleviate anxiety; in contrast, those with insecure attachment styles may find it challenging to engage in supportive peer relationships, potentially exacerbating feelings of isolation and negative emotions (Sun et al., 2025). Consequently, targeted interventions that foster positive peer interactions and enhance emotional intelligence can be crucial for improving the emotional wellbeing of students.

The teacher–student relationship is a critical determinant influencing the emotional wellbeing of medical undergraduates. A positive teacher–student relationship not only enhances students' motivation for learning but also substantially mitigates the prevalence of negative emotions. Empirical studies have shown that teacher support and care bolster students' confidence and sense of belonging, thereby diminishing the likelihood of anxiety and depression. Conversely, negative teacher–student relationships—characterized by conflict—can exacerbate emotional distress and lead to disengagement from the learning process (Chen X. et al., 2023; Burns et al., 2022; Wilkinson and Jones Bartoli, 2021). Students who experience supportive interactions with teachers are more inclined to develop resilience and effective coping strategies, which serve as protective factors against future emotional challenges (Shankland et al., 2024). Consequently, fostering positive teacher–student relationships should be prioritized in educational settings because this can significantly enhance students' emotional health and academic success.

Social networks are pivotal in regulating emotions and influencing the experience of negative emotions. The structure and quality of these networks can substantially affect individuals' emotional responses to stressors. There is a complex relationship between social-media usage and negative emotions. Excessive engagement with smartphones and social-media platforms has been linked to heightened levels of loneliness and anxiety symptoms among student populations (Wang et al., 2021; Nikolic et al., 2023). However, many students maintained contact through social networks to receive peer support during the COVID-19 pandemic, effectively mitigating the negative emotions associated with isolation (Wang et al., 2022; Sundarasen et al., 2020; Cauberghe et al., 2021). This highlights the dual nature of social networks, which can serve as both a source of support and a potential trigger for negative emotional experiences.

### 3.3 Impact of psychological qualities on negative emotions

Psychological resilience is defined as the capacity of individuals to adapt to stress and adversity and recover effectively from challenges; it is integral to the management of negative emotions, particularly in high-stress environments such as medical education. Research suggests that individuals with higher levels of resilience experience fewer negative emotions because they have enhanced capabilities to cope with stressors. For example, a study conducted among medical undergraduates found that those with greater resilience reported lower levels of anxiety and depression, indicating that resilience serves as a protective buffer against negative emotional experiences (Ali et al., 2023). Additionally, resilience has been associated with more-effective emotion regulation strategies, allowing individuals to navigate challenging situations without being overwhelmed by negative emotions (Teh et al., 2023). The relationship between resilience and negative emotions is further corroborated by evidence indicating that resilient individuals tend to use adaptive coping strategies such as problem-solving and cognitive reappraisal, which alleviate the impact of stressors and promote emotional wellbeing (Jay et al., 2023; Thompson et al., 2016; Wu et al., 2020). Masten's resilience framework provide a theoretical basis for resilience-building interventions. Promoting resilience through targeted interventions may decrease substantially the incidence of negative emotions within vulnerable populations, particularly among students experiencing academic stress.

Cognitive styles refer to the preferred methods by which individuals process information and interpret experiences. Research indicates that cognitive styles—especially those associated with emotion regulation—can determine how individuals experience and manage negative emotions (Tsormpatzoudi et al., 2023; Sutton et al., 2011). Cognitive flexibility—defined as the capacity to adapt one's thinking in response to evolving emotional contexts—is crucial for effective emotion regulation. Moreover, studies have shown that cognitive styles are not immutable: they can be modified through experiences and interventions designed to enhance emotional intelligence and coping strategies. For instance, training programs that concentrate on developing cognitive reappraisal skills have been shown to improve emotional outcomes among participants, resulting in reduced negative emotions and enhanced overall psychological wellbeing (Delgado et al., 2023). Therefore, understanding and modifying cognitive styles could be pivotal in managing negative emotions effectively.

### 3.4 Impact of self-management on negative emotions

Effective time management is an essential skill for achieving academic success and maintaining emotional wellbeing among students. Inadequate time management can exacerbate academic pressure, leading to increased stress and negative emotional states. Empirical studies indicate that students who face challenges with time management frequently encounter difficulties in meeting deadlines, which can result in feelings of anxiety and being overwhelmed (Ruan et al., 2024). In contrast, students who use effective time-management strategies typically report lower stress levels and greater satisfaction with their academic performance. The relationship between time management and emotion regulation is multifaceted, and students who manage their time efficiently are better equipped to allocate resources for studying, self-care, and relaxation, thereby mitigating negative emotional experiences (Ding et al., 2024). Moreover, the development of time-management skills can significantly enhance students' perceived control over their academic responsibilities, thereby alleviating feelings of helplessness and anxiety. Research indicates that interventions designed to improve time-management abilities can have a beneficial impact on students' emotion-regulation capabilities. For example, programs that instruct students in task prioritization and realistic goal-setting can lead to improved emotional outcomes because then the students feel more competent in managing academic demands (Wang, 2019). This highlights the importance of incorporating time-management training into academic support services.

Emotion-regulation strategies are crucial mechanisms that individuals use to manage their emotional experiences, especially in response to negative emotions. Gross's Process Model of emotion regulation can effectively explain individual differences in emotional responses and the effectiveness of various coping strategies. The efficacy of these strategies can vary considerably based on the context and the individual's psychological characteristics. Research suggests that adaptive strategies such as cognitive reappraisal and acceptance are generally more effective in alleviating negative emotions than are maladaptive strategies such as suppression and avoidance (Ruan et al., 2024; Wang et al., 2024). Zhang et al. (2025) investigated the emotional responses of medical undergraduates, and found that individuals who used cognitive reappraisal reported significantly lower levels of stress and anxiety compared to their counterparts who relied on suppression. Furthermore, the interaction between academic stress and emotion-regulation strategies is pivotal in determining the degree of negative emotions experienced by students. Those with proficient emotion-regulation skills are better equipped to manage stress and anxiety, whereas individuals lacking these skills may become overwhelmed by academic pressures (Liu et al., 2024). The effectiveness of these strategies is influenced substantially by the context in which they are applied: strategies that are successful in one emotional context may not be equally effective in another. This underscores the critical importance of context-specific interventions designed to enhance individuals' capacity to select appropriate emotion-management strategies tailored to their emotional state and situational demands (Zhang et al., 2022; Mikkelsen et al., 2023).

## 4 Conclusion and future prospects

This review has examined the intricate relationship between negative emotions and medical undergraduates. Our analysis indicates that a range of factors—including academic pressure, interpersonal relationships, psychological traits, and self-management skills—contribute significantly to the prevalence and intensity of negative emotional states among these students. It is crucial to acknowledge that these factors do not function independently but rather interact with one another, creating a unique landscape of mental health challenges that medical undergraduates encounter on a daily basis. Academic pressure—often characterized by high expectations and demanding coursework—can intensify feelings of anxiety and inadequacy. Likewise, the social dynamics inherent in medical education—such as peer competition, the necessity for collaboration, and the development of communication skills—can either bolster or undermine students' emotional wellbeing. Moreover, the individual psychological characteristics that students bring to their medical education—such as resilience, coping strategies, and prior experiences with stress—significantly influence their responses to these pressures. Understanding the interaction of these factors can guide the development of targeted psychological health interventions in medical education.

However, we have identified certain limitations in the current body of research. Many previous studies used cross-sectional designs, which make it difficult to uncover the dynamic mechanisms underlying the evolution of negative emotions. The lack of integration between objective physiological testing indicators and subjective survey questionnaire data restricts the comprehensive analysis of emotion-regulation mechanisms. Consequently, it is essential to balance various research perspectives and findings in this domain. Also, most previous research was conducted in a single cultural context, limiting the generalizability of the conclusions. Future research should incorporate cross-cultural comparisons to examine the differential impact of social values and educational systems on the mental health of medical undergraduates.

In summary, the development of negative emotions among medical undergraduates arises from the interplay of various factors. Addressing this issue necessitates comprehensive and multifaceted approaches that thoroughly consider academic, social, and personal dimensions. Future medical education should prioritize the enhancement of psychological resilience, integrate emotion-management skills into the core competency framework, balance diverse research perspectives, and foster collaboration among all stakeholders to facilitate the holistic physical and mental development of medical professionals.
